# *In vivo* and *in vitro* studies on the roles of neutrophil extracellular traps during secondary pneumococcal pneumonia after primary pulmonary influenza infection

**DOI:** 10.3389/fimmu.2013.00056

**Published:** 2013-03-05

**Authors:** Anandi Narayana Moorthy, T. Narasaraju, Prashant Rai, R. Perumalsamy, K. B. Tan, Shi Wang, Bevin Engelward, Vincent T. K. Chow

**Affiliations:** ^1^Department of Microbiology, Infectious Diseases Program, National University of SingaporeKent Ridge, Singapore; ^2^Center for Veterinary Health Sciences, Oklahoma State UniversityStillwater, OK, USA; ^3^Infectious Diseases Interdisciplinary Research Group, Singapore-Massachusetts Institute of Technology Alliance in Research and TechnologySingapore; ^4^Department of Pathology, National University HospitalSingapore; ^5^Department of Biological Engineering, Massachusetts Institute of TechnologyCambridge, MA, USA

**Keywords:** NETs scoring, neutrophils, primary influenza, secondary pneumonia, *Streptococcus pneumoniae*, mouse models

## Abstract

Seasonal influenza virus infections may lead to debilitating disease, and account for significant fatalities annually worldwide. Most of these deaths are attributed to the complications of secondary bacterial pneumonia. Evidence is accumulating to support the notion that neutrophil extracellular traps (NETs) harbor several antibacterial proteins, and trap and kill bacteria. We have previously demonstrated the induction of NETs that contribute to lung tissue injury in severe influenza pneumonia. However, the role of these NETs in secondary bacterial pneumonia is unclear. In this study, we explored whether NETs induced during pulmonary influenza infection have functional significance against infections with *Streptococcus pneumoniae* and other bacterial and fungal species. Our findings revealed that NETs do not participate in killing of *Streptococcus pneumoniae in vivo* and *in vitro*. Dual viral and bacterial infection elevated the bacterial load compared to animals infected with bacteria alone. Concurrently, enhanced lung pathogenesis was observed in dual-infected mice compared to those challenged with influenza virus or bacteria alone. The intensified NETs in dual-infected mice often appeared as clusters that were frequently filled with partially degraded DNA, as evidenced by punctate histone protein staining. The severe pulmonary pathology and excessive NETs generation in dual infection correlated with exaggerated inflammation and damage to the alveolar-capillary barrier. NETs stimulation *in vitro* did not significantly alter the gene expression of several antimicrobial proteins, and these NETs did not exhibit any bactericidal activity. Fungicidal activity against *Candida albicans* was observed at similar levels both in presence or absence of NETs. These results substantiate that the NETs released by primary influenza infection do not protect against secondary bacterial infection, but may compromise lung function.

## Introduction

Secondary bacterial super-infections are the most frequent complications among fatal cases of seasonal influenza and of pandemic influenza. Primary influenza virus infection predisposing to secondary bacterial pneumonia was highlighted by autopsy analyses during the 1918 influenza H1N1pandemic, as over 95% of deaths were attributed to secondary bacterial infections (Simonsen, 1999; Johnson and Mueller, 2002; Morens et al., 2008; Estenssoro et al., 2010). Bacterial pathogens including *Streptococcus pneumoniae* (*S. pneumoniae*), *Staphylococcus aureus (S. aureus), and Haemophilus influenzae (H. influenzae*) are frequently associated with secondary infections (Kyaw et al., 2006). The incidence of bacterial pneumonia was 25–56% in patients infected with 2009 pandemic swine influenza H1N1 virus, especially severely ill patients (Dominguez-Cherit et al., 2009; Kumar et al., 2009; Palacios et al., 2009; Estenssoro et al., 2010). Similarly, most fatalities due to seasonal influenza are linked to bacterial super-infections (McCullers, 2006; Lei et al., 2010). The extent of influenza-induced cytopathic damage and the magnitude of immune response in the lungs influence the morbidity and mortality in bacterial super-infections. Histopathologically, secondary bacterial infections following influenza exhibit marked inflammation, bronchopneumonia, bacteremia, and diffuse damage in multiple lobes of the lungs (McCullers and Rehg, 2002; McCullers, 2004; Kash et al., 2011). Evidence is accumulating to support that severe outcomes in secondary bacterial complications are due to exaggerated inflammatory responses and neutrophil-predominant infiltrations (Morens et al., 2008; Karlström et al., 2011). Induction of lung inflammatory cytokines (e.g., TNF-α, IL-1β) and chemokines (e.g., mouse KC, MIP-1α) with accompanying intense neutrophil influx within the damaged areas after challenge with *S. pneumoniae* following primary influenza implicates the role of neutrophils in lung pathogenesis (Smith et al., 2007). Although bacterial infections are most common, secondary fungal infections with *Aspergillus* or *Zygomycota* species are also occasionally encountered in severe influenza infections. Invasive zygomycosis was reported in patients who succumbed to pandemic H1N1-2009 virus infection (Guarner et al., 2006; Bal et al., 2012).

Our previous findings demonstrate the participation of neutrophil extracellular traps (NETs) in lung pathogenesis during influenza (Narasaraju et al., 2011). NETs were initially identified for their role in bactericidal activity, and were implicated in innate immunity (Brinkmann et al., 2004; Fuchs et al., 2007). DNA fibers in NETs form web-like structures and harbor several antibacterial proteins that aid in trapping and killing bacteria or other microbial pathogens. However, the prolonged presence of NETs is linked with host tissue damage and risk for development of auto-reactivity against various components in NETs. Furthermore, NETs have been observed in inflammatory, autoimmune, and vascular diseases (Baker et al., 2008; Kessenbrock et al., 2009; Garcia-Romo et al., 2011). The close attachment of DNA strands in NETs carrying cytotoxic proteases leads to thin endothelial damage documented in sepsis and small vessel vasculitis (Garcia-Romo et al., 2011). We previously reported NETs embroiled with thin alveolar-capillary surfaces of the lungs during severe influenza (Narasaraju et al., 2011). NETs-associated proteins including histone and myeloperoxidase (MPO) are directly involved in inducing cytotoxic effects in alveolar epithelial and endothelial cells (Saffarzadeh et al., 2012).

Although NETs generation is evident in bacterial infections such as *S. pneumoniae*, there are hitherto no *in vivo* reports on the characterization of NETs induction during secondary bacterial pneumonia following primary influenza. It is not known whether NETs produced during influenza exacerbates secondary bacterial pneumonia or if they play beneficial roles by trapping and killing bacteria. This study characterized the induction of NETs during secondary bacterial pneumonia following primary influenza. Our findings revealed increased release of NETs during secondary bacterial infection. The presence of significant NETs release during influenza did not reduce bacterial replication in the lungs. Moreover, *in vitro* NETs stimulation did not significantly alter the gene expression of several antibacterial proteins, and these NETs did not exert any bactericidal activity. These results indicate that NETs released during primary influenza do not confer a protective role.

## Materials and methods

### Microorganisms, animals, and ethics approval

Influenza virus A/Puerto Rico/8/34 H1N1 (PR8) obtained from the American Type Culture Collection was propagated in embryonated eggs, and viral titers were determined as described previously (Narasaraju et al., 2011). *S. pneumoniae* serotype 19F was cultured in brain-heart infusion broth supplemented with 5% fetal calf serum under anaerobic conditions. *Klebsiella pneumoniae* K15 (*K. pneumoniae*) was cultured in Luria–Bertani broth under aerobic conditions. For experiments, both bacterial species were harvested at their mid-logarithmic phases at 37°C. Bacterial optical density (OD) was measured at 600 nm, and cell numbers were calculated by growth curves based on OD and standard colony counts of serial dilutions. *S. aureus* and *Pseudomonas aeruginosa* (*P. aeruginosa*) were also employed for *in vitro* experiments to study NETs generation and degradation. All bacterial strains were clinical isolates from Singapore. *Candida albicans* (*C. albicans*, a clinical isolate from Singapore) was cultured on Sabouraud dextrose agar under aerobic conditions at 28°C. Cell numbers were counted with a hemocytometer. Female BALB/c and C57BL/6 mice (7–10 weeks old) were used, and housed in micro-isolator cages in an animal BSL-2 laboratory. All animal protocols (050/11 and 117/10) were approved by the Institutional Animal Care and Use Committee, National University of Singapore.

### Viral and bacterial infections of mice

For all infections, animals were anesthetized with a mixture of 75 mg/kg ketamine and 1 mg/kg medetomidine, and revived with Antisedan (atipamezole hydrochloride) solution given by intraperitoneal injection. C57BL/6 mice were infected intratracheally with lethal doses of influenza PR8 virus, i.e., 250 plaque-forming units (PFU) or ~1 LD_50_. Seven days after influenza infection, mice were challenged with sub-lethal doses of 10^5^ colony-forming units (CFU) of *S. pneumoniae* 19F in 50 μl of phosphate-buffered saline (PBS) via the intratracheal route. Animals infected with bacteria alone also received the same bacterial challenge dose.

### Histopathologic analyses

Lungs from each animal in all groups were fixed in 4% formaldehyde, dehydrated, and embedded in paraffin. Lung sections (4 μm thick) were stained with hematoxylin and eosin. A semi-quantitative histopathologic scoring system was employed in a blinded manner by experienced histopathologists. To generate each lung injury score, multiple fields were examined per slide at 400 × magnification. Within each field, points were assigned according to predetermined criteria as described previously (Matute-Bello et al., 2001). The points for each category were added and weighted according to their relative importance, and the injury score was calculated according to the following modified formula: (alveolar hemorrhage) + 2 × (alveolar infiltrate) + 3 × (fibrin) + (alveolar septal congestion).

### Detection and quantification of NETs in lung sections by immunostaining

Immunohistochemical analyses of formalin-fixed lung sections were performed. Briefly, lung sections were deparaffinized in Histo-Clear, rehydrated through an ethanol series, permeabilized with 0.025% Triton X-100 in Tris-buffered saline (TBS) for 10 min, and blocked with 3% bovine serum albumin in TBS for 1 h. The sections were then incubated overnight at 4°C with 1:1000 dilutions of mouse monoclonal anti-histone H2B antibody and rabbit polyclonal anti-MPO antibody (Abcam). After washing thrice with TBS, the sections were incubated with 1:250 dilutions of anti-mouse Alexa Fluor 488 and anti-rabbit Alexa Fluor 555 secondary antibodies (Molecular Probes), as well as DAPI (Invitrogen) at room temperature for 1 h. The sections were then washed with TBS, mounted with anti-fade mounting medium (Invitrogen), and examined under an IX81 Olympus confocal microscope. All images were processed using the FV10-ASW 3.0 viewer.

For quantification of NETs, the slides were scanned using a high resolution MIRAX MIDI system (Carl Zeiss), and 20 fields per slide were captured randomly at 40 × magnification using the Pannoramic viewer. We designed a scoring system to quantify NETs in lung sections microscopically based on the distribution of structures of NETs (Table 1). To identify NETs, we selected two major morphologic patterns of NETs formation (individual single strands or clusters of DNA) co-stained for MPO, histone H2B, and DAPI, and assigned scores based on their appearance in each field. The areas of the clusters were measured using ImageJ software, and were used as differential criteria to assign scores. The total score was calculated as the sum of scores of 20 fields. For comparison of NETs counts, the mean scores were derived for three groups (*N* = 6 each), i.e., primary influenza, primary bacterial, and secondary bacterial infections.

**Table 1:** **Scoring system for the quantitative evaluation of NETs in the lungs of infected animals**.

**Category**	**Number**	**Area (μm^2^)**	**Score**
None	N.A.	N.A.	0
Single strand	<5	N.A.	1
Single strand	≥5	N.A.	2
Cluster, small	1	<500	2
Cluster, small + single strands/clusters	>1	<500	4
Cluster, medium	1	500–5000	6
Cluster, medium + small clusters/strands	>1	500–5000	8
Cluster, large ± small clusters/strands	N.A.	>5000	10

Total score, sum of scores in 20 fields under 400× magnification. Areas of demarcated NETs clusters were computed using ImageJ software.

N.A., Not applicable.

### Measurement of bacterial load and viral mRNA in murine lungs

Mice were euthanized at 30% weight loss, and the left lobes of lungs were harvested under sterile conditions in liquid nitrogen. The lungs were homogenized in PBS using a gentleMACS dissociator (Miltenyi Biotec). Pneumococcal colony counts of lung homogenates were determined by plating 10-fold serial dilutions on tryptic soy agar plates supplemented with 5% sheep erythrocytes. For quantification of influenza viral nucleoprotein mRNA, real-time RT-PCR was performed using primers 5′-GGGTGAGAATGGACGAAAAA-3′ and 5′-TCCATCATTGCTTTTTGTGC-3′ as described previously (Yamada et al., 2012).

### Isolation of bone marrow-derived neutrophils from mice

Neutrophils were isolated using Percoll-gradient according to a previous protocol (Ermert et al., 2009). Briefly, bone marrow from 7–10-week-old female BALB/c mice was flushed out of the tibia and femur in Dulbecco's PBS without Ca^2+^ and Mg^2+^ (Biowest), homogenized with a 22-gauge needle, and passed through a 70-μm cell strainer to obtain single cell suspensions. Cells were washed once, and layered on discontinuous Percoll gradient, i.e., 78, 69, and 52% Percoll in PBS, and centrifuged for 30 min at 1500 × g. Mature neutrophils were recovered from the interphase between 69 and 78% Percoll. The purity of mature neutrophils was greater than 90% as assessed by modified Giemsa staining.

### Collection of bronchoalveolar lavage fluid (BALF)

BALF was collected from female BALB/c mice infected intratracheally with 500 PFU of PR8 virus (lethal dose) or control PBS (mock infection) at 5 days post-infection (dpi) as described previously (Narasaraju et al., 2011). The BALF was centrifuged, and the aliquots of cell-free supernatants were stored at −80°C until further use.

### Quantification of NETs *in vitro* and effect of inhibitors of redox enzymes on NETs production

We previously demonstrated that NETs generation is induced by redox enzymes such as MPO and superoxide dismutase (SOD) when neutrophils were co-cultured with influenza-infected alveolar epithelial cells (Narasaraju et al., 2011). To substantiate whether redox enzymes released into the alveolar space during infection contribute to NETs formation, we incubated neutrophils isolated from bone marrow of uninfected mice with BALF collected from influenza-infected or mock-infected animals. Neutrophils (0.5 × 10^6^) were incubated with infected BALF for 15 min, 30 min, 1 h, 1.5 h, and 2 h in 8-well chamber slides. At the end of each time-point, the supernatant was removed, the NETs were fixed with formaldehyde, stained, mounted, and observed under fluorescence microscopy. At least 10 fields were captured per well under 400× magnification. NETs were quantified as the percentage of positive events (neutrophils undergoing NETosis) out of total neutrophils in an average of 10 fields (Berends et al., 2010). To determine whether the induced redox enzymes are involved in NETs release, neutrophils were pre-treated for 15 min with 10 μM diphenyleneiodonium chloride (DPI, an inhibitor of NADPH oxidase), 100 μM diethylthiocarbamate (DETC, an inhibitor of SOD), or 100 μM of 4-aminobenzoic hydrazide (ABAH, an inhibitor of MPO) followed by incubation with uninfected or infected BALF for 2 h. NETs were quantified as described above.

### Gene expression of antimicrobial proteins during *in vitro* NETs generation by quantitative real-time RT-PCR

We previously demonstrated that MPO activation induces NETs, and mice challenged with lethal influenza show induced MPO activity in BALF. Incubation of neutrophils with BALF collected from influenza-infected mice significantly induces NETs generation (Narasaraju et al., 2011; Ng et al., 2012). In this study, we evaluated the gene expression of specific proteins during active NETs generation. We selected six proteins associated with bactericidal activity, i.e., cathelicidin, lactotransferrin, pentraxin-3, matrix metalloproteinase-9 (MMP9), S100A8, and S100A9. For NETs induction, neutrophils (1.5 × 10^6^ per well) were incubated with 150 μl of BALF isolated from influenza-infected or mock-infected mice for 15 or 30 min, 1, 1.5, or 2 h. RNA was extracted using the RNeasy RNA purification kit (Qiagen), and reverse-transcribed with MMLV reverse transcriptase (Promega). The resultant cDNAs were subjected to real-time PCR analysis using LightCycler SYBR Green PCR mix (Roche). The following gene primers were employed, i.e., mouse cathelicidin-related antimicrobial peptide (5′-GGCTGTGGCGGTCACTATC-3′ and 5′-GTCTAGGGACTGCTGGTTGAA-3′); pentraxin-3 (5′-CGCAGGTTGTGAAACAGCAAT-3′ and 5′-ATGCACGCTTCCAAAAATCTTC-3′); MMP9 (5′-GCAGAGGCATACTTGTACCG-3′ and 5′-TGATGTTATGATGGTCCCACTTG-3′); lactotransferrin (5′-CCGCTCAGTTGTGTCAAGAAA-3′ and 5′-CATGGCATCAGCTCTGTTTGT-3′); S100A8 (5′-AAATCACCATGCCCTCTACAAG-3′ and 5′-CCCACTTTTATCACCATCGCAA-3′); S100A9 (5′-GCACAGTTGGCAACCTTTATG-3′ and 5′-TGATTGTCCTGGTTTGTGTCC-3′); and GAPDH as the housekeeping gene control (5′-CTTCATTGACCTCAACTACA-3′ and 5′-ATATTTCTCGTGGTTCACAC-3′).

### Antibacterial and antifungal activities, and analysis of NETs degradation and bacterial entrapment

Bactericidal activity of neutrophils was ascertained by co-incubating neutrophils with *S. pneumoniae* or *K. pneumoniae*. Neutrophils (0.5 × 10^6^) were incubated with 150 μl of infected or uninfected cell-free BALF for 1 or 2.5 h to generate NETs. *S. pneumoniae* 19F or *K. pneumonia*e K15 bacteria were added at multiplicity of infection (MOI) of 0.2, 0.1 or 0.01 for 120, 30 or 90 min, respectively. Cytochalasin B (10 μg/ml) was added to each test well 15 min prior to addition of bacteria to inhibit phagocytosis. After incubation, the contents of each well were scraped thoroughly, and serial dilutions were plated onto 5% sheep blood agar (for *S. pneumoniae*) and Luria–Bertani agar (for *K. pneumoniae).* After overnight incubation at 37°C, colony counts were carried out to determine the number of CFU.

Fungicidal activity was ascertained in a similar manner by incubating 0.5 × 10^6^ neutrophils for 2.5 h with infected or control BALF in 0.1% gelatin-coated wells, to which *C. albicans* was added at MOI of 0.1. Cytochalasin B (10 μg/ml) was added to each test well 15 min prior to addition of *C. albicans* to inhibit phagocytosis. After incubation, the contents of each well were scraped thoroughly, and serial dilutions were plated onto Sabouraud dextrose agar. After overnight incubation at 37°C, colony counts were performed to determine the number of CFU.

To ascertain whether bacteria can cause degradation of NETs DNA, we incubated 0.5 × 10^6^ neutrophils with *S. pneumoniae, K. pneumoniae, S. aureus*, and *P. aeruginosa* at MOI of 1. After incubation for 2 h, NETs were stained with DAPI and observed under fluorescence microscopy. In addition, NETs were induced with infected BALF, and *S. pneumoniae* was added at MOI of 0.01, 0.1, 1, 10, 100, and incubated for 2 h. NETs were quantified as described previously. To visualize bacterial entrapment in NETs, bacteria were stained with a rabbit polyclonal antibody against *S. pneumoniae* 19F for 45 min at room temperature and counterstained with Alexa Fluor 555 for 1 h at room temperature. Bacterial entrapment in NETs was evaluated as the percentage of total NETs showing bacterial entrapment in an average of 10 fields (Berends et al., 2010).

### Statistical analyses

Statistical analyses were performed using Student's *t*-test for pairwise comparison, and ANOVA with Tukey *post-hoc* correction for comparison of more than 2 groups. Non-parametric data were analyzed using Kruskal–Wallis test with *post-hoc* Mann–Whitney pairwise comparison and Bonferroni correction. A value of *P* < 0.05 was considered statistically significant. The data were expressed as mean ± SE.

## Results

### Synergistic effects of primary influenza and secondary bacterial infection on microbial replication and lung pathogenesis

**Figure 1:**
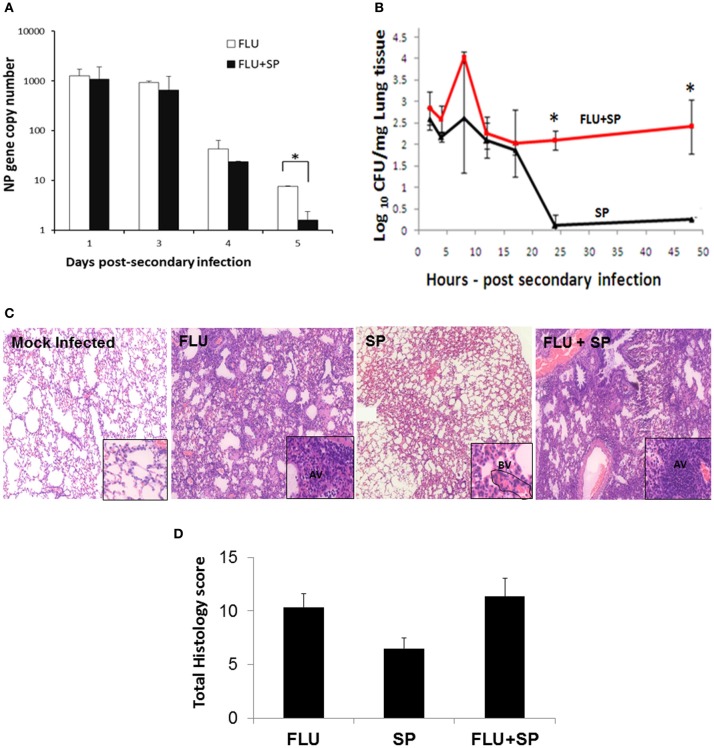
**Effects of influenza and/or *S. pneumoniae* infections on their replication and on lung pathology. (A,B)** C57BL/6 mice were infected with sub-lethal doses (30 PFU) of influenza virus (FLU) or mock-infected with PBS (control). Seven days after influenza infection, mice were challenged with sub-lethal doses of *S. pneumoniae* 19F for viral and bacterial co-infection experiments (FLU + SP). For bacterial infection alone (SP), mice were infected with sub-lethal doses of *S. pneumoniae* 19F. *N* = 3 per animal group. ^*^Denotes *P* < 0.05. **(A)** Expression of the viral nucleoprotein gene was measured by real-time RT-PCR analysis until 5 days post-secondary infection. **(B)** Growth of *S. pneumoniae* in the lung homogenates of mice with dual infection and bacterial infection only was determined by the number of CFU until 48 h post-secondary infection. **(C,D)** C57BL/6 mice were infected with lethal doses of influenza virus (250 PFU) or mock-infected with PBS (control). Seven days after influenza infection, mice were challenged with sub-lethal doses of *S. pneumoniae* (FLU + SP). Influenza infection alone (FLU). *S. pneumoniae* challenge only (SP). **(C)** Histopathologic analyses reveal alveolar septal congestion and inflammation in the lungs of influenza-infected mice, while infection with bacteria alone for 48 h did not cause severe pathology. Dual infection culminated in extensive pulmonary damage and severe pneumonia. Mock-infected animals showed normal alveolar architecture. Mice infected with influenza only had severe inflammation in the alveoli, whereas the inflammation was mainly found in blood vessels (BV) in animals infected with bacteria alone. Dual-infected mice demonstrated severe inflammation in the alveoli (AV). Magnification of 10× (main panels) and 40× (inserts). **(D)** Histopathology scores of lungs showing the mean values ± SE of 6 animals per group. The *P*-values were not significant between the groups.

To determine the synergistic effects of primary influenza and secondary bacterial pneumonia, mice were challenged with lethal doses of influenza, followed by sub-lethal *S. pneumoniae* infection. The effects of co-infection on microbial replication in the lung micro-environment were determined. Interestingly, co-challenge with *S. pneumoniae* generally did not alter virus replication, and viral RNA levels declined similar to animals infected with virus alone. However, significant reduction of virus titers in dual-infected animals was found at 5 dpi (Figure 1A). In contrast, dual infection enhanced bacterial multiplication significantly higher than the animals infected with bacteria alone, indicating the additive effect of influenza on bacterial load (Figure 1B). Influenza infection alone exhibited inflammation predominantly with neutrophils and macrophages, while sub-lethal infection with *S. pneumoniae* alone produced relatively mild inflammatory responses. Infection with bacteria alone for two days did not cause severe pathology: alveolar septal congestion was similar to influenza-infected animals; focal neutrophil-specific cellular infiltrations were observed in the alveolar air spaces, but excessive neutrophils were found within the capillaries and small blood vessels. Co-infection with influenza and *S. pneumoniae* culminated in multi-lobular pulmonary damage compared to infection with virus or bacteria alone. Alveolar air spaces were filled with proteinaceous material and fibrin deposition in dual-infected lungs. The increased inflammatory cell recruitment in dual-infected mice is likely to be driven by elevated levels of lung cytokines and chemokines due to influenza infection prior to bacterial challenge. Influenza infection alone intensified alveolar septal congestion, with mild-to-moderate inflammation mainly with macrophages and neutrophils (Figure 1C and Table 2). No significant difference in overall histopathology score was found between influenza only and dual infection groups, although there was heightened inflammation and tissue consolidation in dual-infected animals. However, the histopathology score was the lowest in mice sub-lethally infected with *S. pneumoniae* (Figure 1D).

**Table 2:** **Histopathology and NETs scoring of lungs of infected animals**.

**Infection group**	**Animal number**	**Alveolar septal consolidation**	**Alveolar hemorrhage**	**Intra-alveolar fibrin**	**Cellular infiltrates**	**Total histo-pathology score**	**Total NETs score**
Influenza only	23	2	2	1	2	7	35
	24	2	1	3	4	10	28
	25	2	1	3	2	8	17
	31	3	1	6	6	16	31
	32	2	1	3	4	10	21
	33	3	1	3	4	11	35
*Streptococcus pneumoniae* only	29	2	0	0	0	2	33
	30	2	1	0	0	3	16
	7.9	2	0	0	0	2	23
	37	2	1	0	0	3	33
	38	2	0	0	2	4	36
	7.10	2	1	0	0	3	35
Influenza + *Streptococcus pneumoniae*	26	2	2	6	6	16	61
	27	2	1	0	2	5	22
	28	2	1	3	3	9	54
	34	2	1	3	4	10	36
	35	2	1	6	6	15	39
	36	2	1	6	4	13	71

### Quantification of increased NETs release in mice challenged with *S. pneumoniae* following primary influenza

**Figure 2:**
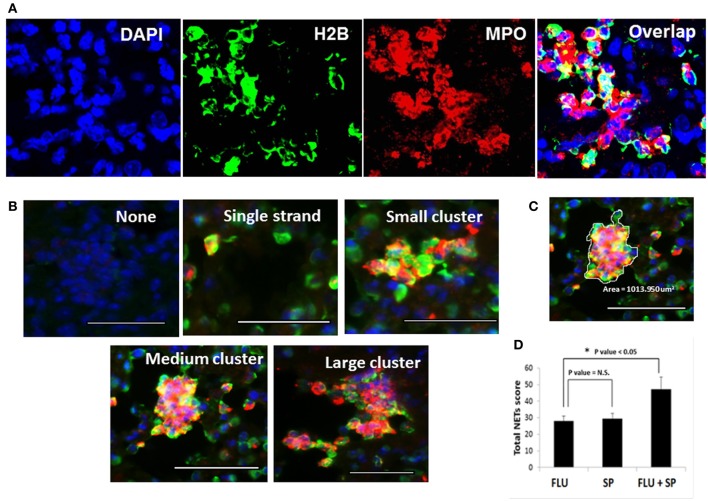
**Detection and quantification of NETs in the lungs of infected mice.** Detection of NETs induced in lung tissues of mice infected with lethal doses of influenza virus followed by *S. pneumoniae* challenge. **(A)** For identification of NETs, lung sections were stained with histone H2B (green), MPO (red), and DAPI (blue). **(B)** NETs formation was analyzed microscopically based on triple immunostaining, and by their morphologic characteristics, i.e., appearing as individual NETS, small, medium, or large clusters of NETs. All scale bars represent 50 μm. **(C)** A representative cluster with area demarcated for calculation by ImageJ software. **(D)** Quantification of the total NETs score was obtained from at least 20 fields of each lung section. Values represent the means ± SE of 6 animals per group. ^*^Indicates *P* < 0.05; N.S. = Not significant difference.

We previously demonstrated that during influenza infection, NETs are induced predominantly in areas of tissue damage. Enhanced NETs release and DNA fibers of the NETs entangled with the alveolar epithelium and small blood vessels are observed in lethal influenza infection (Narasaraju et al., 2011). To determine whether secondary bacterial challenge influences NETs induction, we evaluated the NETs release during primary infections with influenza or *S. pneumoniae* alone, and during dual infection. NETs were identified by triple labeling with histone H2B, MPO, and DAPI (Figure 2A). We performed quantification of NETs in the lung tissues based on the appearance of NETs as individual, small, medium, or large clusters as depicted in Figure 2B. The system for scoring of the unique morphologic structures is indicated in Table 1, while the cluster area calculation is exemplified in Figure 2C. At least 20 fields were counted for each mouse lung under 40× magnification. Dual-infected animals displayed prominently increased and large clusters of NETs in their alveoli and small airways. In contrast, the lungs of mice infected with bacteria or influenza virus alone displayed mainly individual NETs or small clusters of NETs. Prominent staining of MPO and histone in the clusters indicated that these structures were formed mainly by neutrophils. The average total NETs scores were calculated, and revealed that NETs induction was significantly intensified in the dual infection group (Figure 2D) compared to the primary influenza group.

### Degradation of NETs in the presence of *S. pneumoniae* both *in vivo* and *in vitro*

**Figure 3:**
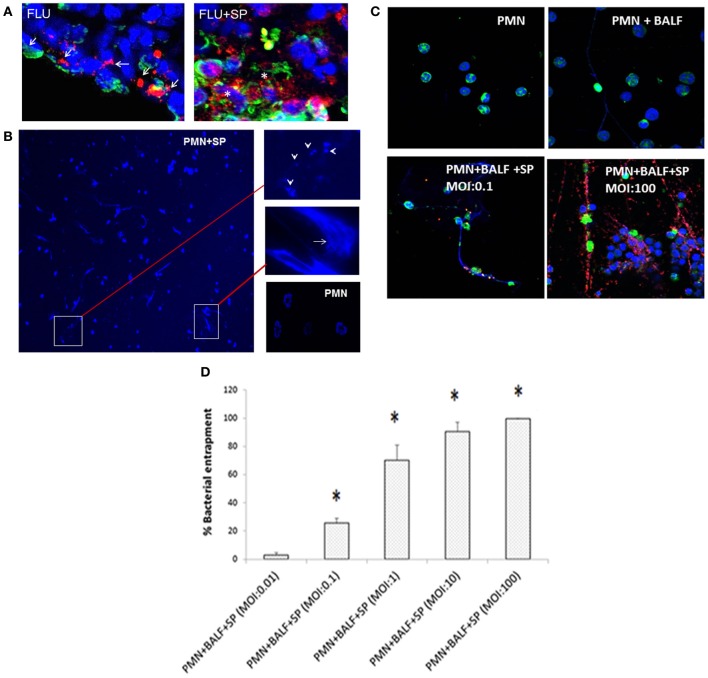
**Partial degradation of NETs and bacterial entrapment within NETs associated with *S. pneumoniae* 19F. (A)** Lungs of mice infected with influenza alone (FLU) exhibit continuous staining (arrows) of MPO (red) and histone H2B (green). However, lungs of mice with viral and bacterial co-infection (FLU + SP) revealed large clusters of NETs often filled with degraded DNA, appearing as punctate staining (asterisks) of MPO and histone H2B. **(B)** To ascertain whether *S. pneumoniae* and other bacterial species cause NETs degradation, neutrophils were isolated from the lungs of influenza-infected mice (Narasaraju et al., 2011), incubated with *S. pneumoniae, K. pneumoniae, P. aeruginosa*, or *S. aureus* at a neutrophil:bacterial ratio of 1:10, and stained with DAPI. Extensive NETs were notable when neutrophils were incubated with *S. pneumoniae* (PMN + SP), compared with incubation with the other 3 bacterial species (data not shown). NETs were negligible for the untreated neutrophils (PMN) serving as negative control. Partially degraded NETs were observed when incubated with *S. pneumoniae*, suggesting DNA degradation in NETs by endonucleases produced by *S. pneumoniae*. Arrow indicates bacteria trapped inside the NETs, while arrowheads depict partially degraded NETs. **(C)** Experiments were performed to study NETs interactions with bacteria. Normal mouse bone marrow-derived neutrophils (PMN) served as negative control. These neutrophils were also incubated with BALF from influenza-infected mice (PMN + BALF). Neutrophils with infected BALF were also incubated with *S. pneumoniae* at various MOI (PMN + BALF + SP). Immunostaining for NETs and bacteria was performed for tests and controls. *S. pneumoniae* bacteria (red) were found entrapped within the DNA fibers (blue) and histone H2B (green) depending on MOI. Higher MOI of 100 displayed greater accumulation and entrapment of bacteria in the vicinity of NETs compared to lower MOI of 0.1. **(D)** Bacterial entrapment on NETs was significantly enhanced with increasing MOI. Values represent the means ± SE of at least 3 independent experiments. ^*^Denotes *P* < 0.05 vs. MOI of 0.01.

**Figure A1:**
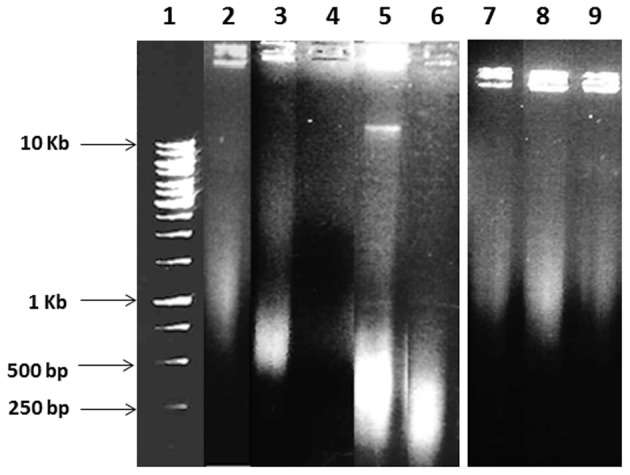
**Analysis of degradation of salmon sperm DNA by *S. pneumoniae* and *K. pneumoniae*.** Salmon sperm DNA (2 μg) was treated with either bacterial pellet (10^7^ cells) or double-filtered supernatant (3 μl) of *S. pneumoniae* 19F or *K. pneumoniae* K15 at 37°C for 1 h in DNase buffer (10 mM Tris, 3 mM MgCl_2_, 5 mM CaCl_2_, pH 7.4), and the reaction was stopped with 0.5 M EDTA. The samples were then subjected to 1% agarose gel electrophoresis. Lanes: (1) DNA ladder. Salmon sperm DNA was incubated with (2) DNase buffer; (3) DNA alone; (4) BHI broth; (5) *S. pneumoniae* pellet; (6) *S. pneumoniae* supernatant; (7) LB broth; (8) *K. pneumoniae* pellet; and (9) *K. pneumoniae* supernatant. The DNA smearing below 500 bp in lanes 5 and 6 revealed that salmon sperm DNA was digested by both pellet and supernatant of *S. pneumoniae*, indicating secreted DNase activity. However, *K. pneumoniae* did not digest the DNA as shown by relatively intact salmon sperm DNAs.

**Figure A2:**
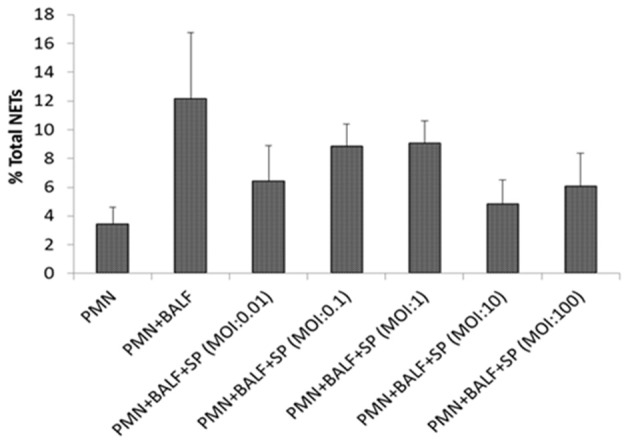
**Quantification of NETs degradation by *S. pneumoniae in vitro*.** Mouse bone marrow-derived neutrophils (PMN) served as negative control. Neutrophils were stimulated for 2 h with BALF from influenza-infected mice (PMN + BALF), which were then incubated with *S. pneumoniae* 19F (PMN + BALF + SP) at MOI of 0.01, 0.1, 1, 10, and 100 for 2 h. After incubation, the remaining NETs were quantified as previously described. Compared with the PMN + BALF group, infection with different MOI of bacteria resulted in modest reduction of total NETs, implying only partial degradation of NETs by *S. pneumoniae* 19F. Values represent the means ± SE of at least 3 independent experiments. The *P*-values were not significant between the PMN + BALF + SP groups versus the PMN + BALF group.

The large clusters of NETs found in dual-infected murine lungs, exhibited typical bundles of NETs with elongated DNA fibers. However, we also noted degrading NETs, as shown by scattered staining of histone protein within these clusters, which also closely stained for MPO (Figure 3A). In contrast, influenza infection alone displayed predominantly continuous staining for histone or MPO. These results indicate partial degradation of NETs in the presence of *S. pneumoniae*, which is known to produce endonucleases that aid the bacteria to escape from being trapped inside the NETs (Beiter et al., 2006; Buchanan et al., 2006). To ascertain whether neutrophils recruited during pulmonary influenza infection produce NETs when they encounter invading bacteria, we incubated neutrophils isolated from influenza-infected mice with different bacteria. Incubation of neutrophils with *S. pneumoniae* resulted in extensive NETs (Figure 3B), compared to incubation with *S. aureus, P. aeruginosa*, or *K. pneumoniae* (data not shown). Bacteria entrapped within the NETs were also observed. Furthermore, partial degradation of NETs with disconnected and punctate staining for DAPI was observed in neutrophils incubated with *S. pneumoniae* (Figure 3B, arrowheads). To assess the interaction of NETs with pneumococci, we incubated NETs (induced by BALF from influenza-infected mice) with bacteria at different MOI. Augmented bacterial entrapment was observed with increasing MOI (Figures 3C,D). To investigate bacterial DNase activity, salmon sperm DNA was incubated with bacterial pellets and supernatants, which revealed that *S. pneumoniae* possessed potent DNase activity, whereas *K. pneumoniae* did not (Figure A1). Incubation of neutrophils with influenza-infected BALF and *S. pneumoniae* at different MOI also resulted in some reduction of total NETs, indicating partial degradation of NETs by *S. pneumoniae* (Figure A2). Taken together, these results suggest the degradation of NETs by pneumococci as an evasion strategy.

### Redox enzymes induced during influenza infection contribute to NETs formation

**Figure 4:**
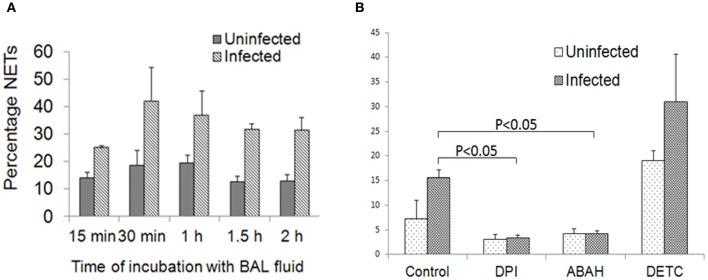
**Redox enzymes mediate NETs induction during influenza infection. (A)** Neutrophils isolated from bone marrow of uninfected mice produced NETs when stimulated with BALF collected from influenza-infected animals. NETs were identified as early as 30 min after incubation. The *P*-values for the various time-points were not significant. **(B)** Incubation of neutrophils with inhibitors of redox enzymes revealed significant reduction in NETs formation in the presence of DPI and ABAH only. Values represent the means ± SE of at least 3 independent experiments. *P* < 0.05 vs. control incubated with infected BALF.

We observed that NETs were generated to a greater degree when neutrophils were incubated with influenza-infected BALF (Figure 4A). Upregulation of several cytokines, chemokines, and redox enzymes is observed during influenza infection. We previously identified that redox enzymes produced by infected epithelial cells stimulate NETs release (Narasaraju et al., 2011). To confirm whether the redox enzymes present in BALF potentially contribute to NETs formation, we incubated neutrophils in the presence of inhibitors of MPO, NADPH oxidase, and SOD. We found significant inhibition of NETs release by MPO and NADPH oxidase inhibitors, but not with SOD inhibitor (Figure 4B). These findings confirmed our previous findings that redox enzymes play a significant role in NETs generation during influenza pneumonia.

### Expression of antimicrobial genes in neutrophils during NETs formation

We previously showed that NETs induction during influenza is regulated by MPO activity, with increased MPO activity found in BALF from animals with lethal influenza. Incubation of neutrophils with BALF collected from influenza-infected mice significantly induced NETs generation. We performed kinetics of NETs release during incubation with BALF, which revealed intensified NETs release with incubation time (Figure 4A). Incubation of neutrophils with BALF from uninfected mice or from mice challenged with sub-lethal influenza generated less NETs. These studies underscore the notion that NETs are induced in the lung micro-environment in the presence of enzymes such as MPO (Narasaraju et al., 2011). We evaluated the gene expression of 6 selected antimicrobial proteins that are associated with NETs, which were identified by DNA microarray analysis of lungs of mice challenged with lethal influenza. Incubation of influenza-infected BALF with neutrophils did not significantly alter the gene expression of cathelicidin, S100A8, S100A9, lactotransferrin, pentraxin-3, and MMP9 (data not shown).

### NETs do not exert bactericidal activities but still possess antifungal properties

**Figure 5:**
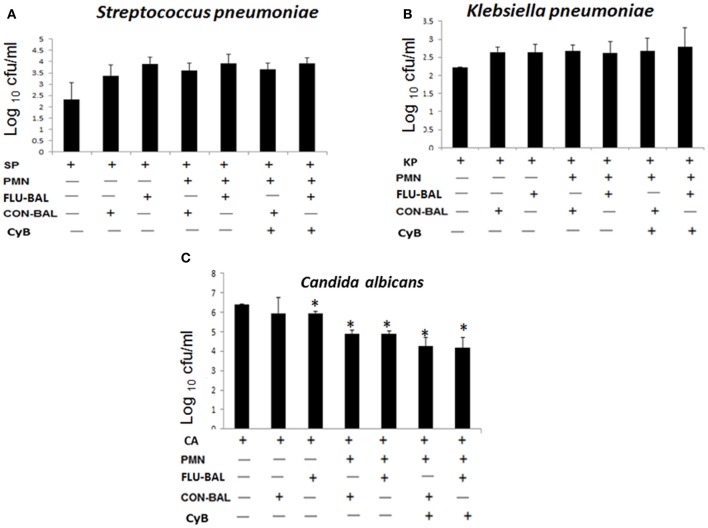
**Bactericidal and fungicidal activities of neutrophils and NETs *in vitro*.** To determine the antimicrobial activity of NETs, neutrophils (PMN) were incubated with virus-infected BALF (FLU-BALF) or uninfected control BALF (CON-BALF) in the presence of **(A)** *S. pneumoniae* 19F or SP, **(B)** *K. pneumoniae* K15 or KP, and **(C)** *C. albicans* or CA with or without cytochalasin B (CyB). After incubation for 2.5 h, the remaining bacteria were plated on sheep blood agar or Luria–Bertani agar, and colonies were counted 24 h later. For colony counts of *C. albicans*, overnight incubation was carried out, followed by plating on Sabouraud dextrose agar. Values represent the means ± SE of at least 3 independent experiments. ^*^Denotes *P* < 0.05 vs. untreated *C. albicans* only control.

To determine whether NETs induced during influenza can kill bacteria, we incubated neutrophils (stimulated with influenza-infected BALF) with *S. pneumoniae* or *K. pneumoniae* in the presence of cytochalasin B (an inhibitor of phagocytic activity). The generation of NETs was more prominent when neutrophils were incubated with *S. pneumoniae* compared to *K. pneumoniae.* However, these NETs did not possess any bactericidal activity (Figures 5A,B). Although invasive fungal infections are not commonly associated with influenza infections, the expression of proteins such as S100A8, S100A9 may also exert antifungal effects. Hence, we investigated whether the NETs induced during influenza possess antifungal activity against *C. albicans*. As shown in Figure 5C, we found significant difference in antifungal activity in the presence of NETs compared with the mock control without neutrophils. However, neutrophils incubated with influenza-infected BALF revealed antifungal effects similar to those neutrophils incubated with uninfected BALF.

## Discussion

Secondary bacterial infection with *S. pneumoniae* was the most predominant critical complication in the 2009 swine influenza H1N1 pandemic (Palacios et al., 2009; Isais et al., 2010; Rice et al., 2012). Although bacterial super-infections have been recognized since the 1918 influenza pandemic, the synergism between influenza and bacterial co-pathogens, and the detailed pathogenic contributions of dual infections to increased morbidity and mortality are not completely understood. Our previous studies linked excessive neutrophil recruitment and release of NETs with lung damage in mice challenged with lethal influenza (Narasaraju et al., 2011). NETs have been characterized for their role in bacterial killing (Brinkmann et al., 2004; Fuchs et al., 2007). In this study, we investigated whether NETs induced during primary influenza influence secondary bacterial replication and pathologic events in the lungs. Our findings revealed that prior challenge with influenza augmented bacterial growth compared to bacterial infection alone. Extensive NETs were prominent during dual infection, which revealed severe pulmonary pathology and tissue consolidation. Furthermore, we observed partially degraded NETs in the lungs of dual-infected mice, as well as when neutrophils were incubated with *S. pneumoniae in vitro*. There were no significant changes in gene expression of several proteins associated with NETs upon stimulation of neutrophils with BALF acquired from influenza-infected mice, and these NETs failed to exhibit any bactericidal activity. Taken together, our studies revealed that NETs induced during influenza infection do not participate in bacterial killing, but may exacerbate lung pathology during secondary bacterial pneumonia.

The higher mortality rates in bacterial super-infections following influenza are associated with increased respiratory failure, inflammation, and bacteremia. The lethal synergism of dual infections varies with the challenge dose of virus or bacteria, and the duration of infections (McCullers and Rehg, 2002; Smith et al., 2007). In our current model, we analyzed lethal influenza, followed by sub-lethal *S. pneumoniae* challenge, because we found extensive NETs generation only during lethal influenza challenge. Although bactericidal effects of NETs are well-explored, most studies have been confined to *in vitro* experiments (Brinkmann et al., 2004; Young et al., 2011; Marin-Esteban et al., 2012). We evaluated the *in vivo* scenario of the pre-existing NETs within the lung micro-environment at the time of bacterial challenge. Despite the presence of excessive NETs in dual-infected mice, there was augmented bacterial replication, suggesting that the NETs released in the lungs are not involved in clearance of *S. pneumoniae.* This study warrants future investigations to explore the effects of NETs on other bacterial pathogens, since *S. pneumoniae* is known to generate endonucleases that can digest NETs. We designed a scoring system for the quantification of NETs based on their appearance in the lungs. Clusters formed due to excessive neutrophils, and NETs released within the damaged areas were more frequently noted in dual-infected animal lungs, while individually formed NETs were predominant in mice infected with influenza alone. Although MPO and histone proteins strongly stained within the clusters, we could not exclude the presence of other cellular debris. Interestingly, DNA fibers in large clusters of NETs in the lungs of dual-infected mice appeared partially degraded, as evident from the punctate appearance of histone and MPO. Degradation of NETs may be mediated by endonucleases that are secreted by *S. pneumoniae*. This phenomenon was further supported by our *in vitro* experiments demonstrating that incubation of neutrophils isolated from influenza-infected mice with *S. pneumoniae* led to partial NETs degradation. Degradation of NETs releases their associated proteins consisting mainly of histones, elastase, and other proteins. Extracellular histones released by either NETs or dying cells can potentially contribute to inflammation and tissue injury during sepsis and other inflammatory responses (Warr and Jakab, 1979; Xu et al., 2009; Saffarzadeh et al., 2012). Histones released by degraded NETs can inflict cytopathic effects in alveolar epithelial and endothelial cells mediated through toll-like receptor-mediated signaling, e.g., TLR2, TLR4 (Kessenbrock et al., 2009; Small et al., 2010; Xu et al., 2011). The results suggest the potential role of NETs and their degrading components in enhanced immunopathology observed during secondary bacterial pneumonia following influenza, and warrants further investigations into the role of NETs in lung injury.

Dual-infected mice suffered greater lung damage compared to influenza infection alone. Sub-lethal *S. pneumoniae* infection alone displayed relatively mild pathology compared with the influenza-infected group. The accumulation of neutrophils and NETs was more obvious in blood vessels rather than air spaces in bacterial infection alone. Although heightened bacterial colonization was observed in dual infection, the influenza virus titers did not change up to 3 days post-bacterial infection, but decreased thereafter possibly due to viral clearance. These results are congruent with previous findings that co-infection of bacteria as early as 2 days after influenza infection does not enhance viral replication, but increases bacterial colonization (Kash et al., 2011). There are possible explanations for the enhanced bacterial loads in dual-infected animals. Firstly, prior damage by influenza exposes the cellular basement membrane to allow the bacteria to disseminate deeper into the lungs. Furthermore, platelet-activating factor receptor (PAFr) is enhanced during influenza infection, which facilitates bacterial attachment to the cell membrane. Blocking PAFr significantly abrogates bacterial multiplication (McCullers and Rehg, 2002). Our data indicated that the presence of NETs did not influence bacterial replication, but may indirectly provide an optimal environment for bacterial growth by increasing alveolar-capillary damage. To substantiate the *in vivo* evidence supporting the lack of bactericidal activity of NETs, we stimulated NETs *in vitro* in the presence of BALF acquired from mice with lethal influenza. We found extensive NETs induction, but there were no significant alterations in gene expression of several antimicrobial proteins during NETs formation. These NETs failed to suppress bacterial growth, and exhibited no significant difference in fungal replication when compared to neutrophils stimulated with uninfected BALF. The lack of antibacterial activity of NETs may be due to the relatively low amount of antibacterial proteins in NETs, and whether these proteins are still active after NETs release. Nevertheless, these results concur with a recent report that NETs can trap bacteria and other pathogens, but may not kill these microorganisms (Menegazzi et al., 2012).

In conclusion, these studies demonstrate that secondary *S. pneumoniae* infection following primary influenza intensified NETs generation and lung pathogenesis. NETs did not participate in bacterial killing as evidenced by both *in vivo* and *in vitro* experiments. Infection with *S. pneumoniae* may cause partial degradation of NETs, which could in turn release NETs-associated proteins, thus contributing to the pulmonary pathology in dual infection.

### Conflict of interest statement

The authors declare that the research was conducted in the absence of any commercial or financial relationships that could be construed as a potential conflict of interest.
